# Prevalence and factors associated with use of khat: a survey of patients entering HIV treatment programs in Ethiopia

**DOI:** 10.1186/s13722-016-0069-2

**Published:** 2017-01-06

**Authors:** Alan R. Lifson, Sale Workneh, Tibebe Shenie, Desalegn Admassu Ayana, Zenebe Melaku, Lemlem Bezabih, Hiwot Tekle Waktola, Behailu Dagne, Rose Hilk, Ken C. Winters, Lucy Slater

**Affiliations:** 1 Division of Epidemiology and Community Health, University of Minnesota, 1300 S. Second Street, Suite 300, Minneapolis, MN 55454-1015 USA; 2 Ethiopian Office, National Alliance of State and Territorial AIDS Directors, Addis Ababa, Ethiopia; 3 College of Health and Medical Sciences, Haramaya University, Dire Dawa, Ethiopia; 4 International Center for AIDS Care and Treatment Program, Addis Ababa, Ethiopia; 5 Oregon Research Institute, Eugene, OR USA; 6 Global Program, National Alliance of State and Territorial AIDS Directors, Washington, DC USA

**Keywords:** HIV, Khat, Amphetamine, Sub-Saharan Africa, Ethiopia

## Abstract

**Background:**

Khat, a plant native to East Africa, has psychoactive constituents similar to amphetamine. Chronic khat use can lead to psychological dependence with multiple physical and mental health harms, complicating clinical management of people living with HIV. In two Ethiopian cities where khat is common, we evaluated prevalence and correlates of khat use among patients new to HIV care.

**Methods:**

During 2013–2014, we surveyed 322 patients recently enrolled in HIV clinics in Dire Dawa and Harar about khat use, demographics, smoking and alcohol use, clinical illness, food insecurity, and social support. We analyzed factors associated with khat use in the past year, as well as heaviest use of khat (based on greatest number of hours used in a typical month).

**Results:**

242 (75%) respondents reported lifetime khat use; 209 (65%) reported khat use during the previous year. 54% of khat users started before age 19 years. Although 84% believed that using khat every day is dangerous for health if you have HIV, khat was used in the previous year a median of 5 h/days and 30 days/month; 21% said they felt a need to cut down or control their khat use but had difficulty doing so. Those using khat were more likely to report smoking (46%) and alcohol use (49%) compared to non-khat users (1 and 31% respectively). Those reporting heaviest khat use (≥180 h/typical month) were more likely to rate their health status as poor, have an underweight BMI (≤18.5 kg/m^2^), report more symptoms of chronic illness, and agree with more statements indicating a negative physical quality of life. In multivariate analysis, heavy users were more likely to be male, Muslim, and non-married.

**Conclusions:**

Khat use was common among HIV patients entering care, and associated with symptoms of poorer physical health. Over half started khat use when they were young. Although most believed khat is harmful for HIV patients, a number of respondents reported some difficulty controlling their drug use. In settings where khat is legal and widely utilized, developing interventions for responsible use represent an important health priority as part of comprehensive care for people living with HIV.

## Background

Khat, or *Catha edulis*, is a flowering plant native to East Africa which is legal to grow, sell and consume in Ethiopia and used for its stimulant effects [1–3]. Most often the fresh leaves and shoots are chewed, although it can also be made into a tea or paste. The psychoactive constituents of khat, cathinone and cathine, are structurally similar to amphetamine. Immediate effects of khat may include euphoria, feelings of increased alertness and energy, hyperactivity, appetite suppression, and lack of fatigue. Acute psychological effects of khat use may include paranoia, hyperactivity, mania, withdrawal or isolation, and aggressive behavior [1–3]. Chronic khat use can lead to psychological dependence, manic behavior, violence, psychosis and other mental disorders [1, 3, 4]. Physical consequences of khat use can include hypertension and cardiovascular disease [2, 3, 5].

Khat use has been described as common in many Ethiopian populations [6–12]. A household survey of 1200 adults from one rural Ethiopian community found that 32% were khat users [6]. One survey of youth 15–24 years found 38% used khat in the previous year [12], while another study of high school students found 24% were khat chewers [9]. A survey of a representative sample of adults from throughout Ethiopia found wide geographic disparities in prevalence of khat use; in one high-prevalence community (Dire Dawa), 52% reported a lifetime history of khat use, of whom 58% reported use every day during the previous year [7].

Ethiopian studies show a relationship between khat use and unsafe sex, which may predispose to human immunodeficiency virus (HIV) infection [7, 12, 13]. Ethiopian khat users were more likely to have multiple sexual partners, to have sex with a non-regular partner, to exchange sex for money and to report a history of a genital ulcer [7, 12]. In one case-control Ethiopian study, khat use was associated with a twofold increased risk of HIV infection [13].

Despite the suggestion of significant overlap between the khat and HIV epidemics in Ethiopia, there are very limited data on prevalence and patterns of khat use in people living with HIV. This is particularly important since studies suggest that khat users may have poorer adherence to HIV therapy [7, 11], complicating their medical management. In addition to the physical and mental health harms that may be associated with khat, khat use has also been associated with an increased prevalence of smoking and alcohol use [10, 11], each of which can have their own adverse health effects for people with HIV [14, 15].

During 2013–2014, we enrolled patients in a 12 month prospective study from HIV clinics in two Ethiopian cities where khat use is especially common. Because we were interested in evaluating the impact of khat use on retention in clinical care, we chose those patients who had recently (within the last 3 months) enrolled in HIV care. Studies from Ethiopia and elsewhere show that loss to follow-up may be greatest within the first 6 months after entering care [16, 17], and it is patients new to care who are often most clinically and psychologically vulnerable.

We report results from our baseline survey evaluating prevalence and correlates of khat use in the previous year among patients newly enrolled in HIV clinics. Because khat use was relatively common in this population, we also analyzed factors associated with the heaviest use of khat, based on those with the greatest number of hours khat was used in a typical month during the preceding year.

## Methods

### Study setting

Adult patients (≥18 years) newly enrolled in HIV care (within previous 3 months) were identified and sequentially offered enrollment at two HIV clinics: Dil Chora regional hospital in Dire Dawa, and Hiwot Fana hospital (operated by Haramaya University) in Harar. Dire Dawa is a city located in eastern Ethiopia, with an estimated 2012 population of 262,884 [18], and is a major commercial and industrial hub. Harar is an ancient city and cultural heritage site in the highlands of eastern Ethiopia, with an estimated 2012 population of 110,457 [18]. Dire Dawa and Harar were selected as two cities with large HIV clinics, and where khat is commonly grown and used. Data from the Ethiopian Demographic and Health Survey reported an HIV seroprevalence of 4.0% in the Dire Dawa region and 2.8% in the Harari region [19]. In the same survey, although 11% of all Ethiopian women reported ever chewing khat, rates were 27% and 39% in the Dire Dawa and Harari regions, respectively [19]. Similarly, 28% of Ethiopia men reported ever chewing khat, but in the Dire Dawa and Harari regions, these rates were 79 and 82% respectively [19].

### Data collection

A survey verbally administered in Amharic to participants included assessment of: (a) demographic characteristics, (b) khat use and attitudes, (c) smoking and alcohol use, (d) clinical illness, (e) food insecurity, and (f) social support. Survey items were adapted from other surveys plus our previous questionnaires with Ethiopian HIV patients [20–22]. Questions were translated into Amharic, and then pilot tested to ensure adequacy of translation, clarity, and cultural appropriateness.

Khat use questions included lifetime use and use within the previous year; for those reporting use in the prior year, we asked about days used in a typical month, and hours used in a typical day. To quantify khat use in the past year, we created summary scores of typical hours used per month, defined as the product of typical hours used per day and typical days used per month. We defined heavy khat use as the top quartile (greatest use) of typical hours used per month. We asked whether participants felt the need to cut down or control their khat use but had difficulty doing so; we also asked whether they used khat within a half hour after waking, used it in places one shouldn’t (e.g., church, work), and used it even if they were sick in bed.

We inquired about and quantified use of alcohol and tobacco. Those who reported drinking in the past year were asked how many days they drank alcohol during a typical month, and how many drinks they took in a typical day when they did drink; the number of drinks during a typical month was then calculated as the product of drinks/day and days/month.

Severity of clinical illness was assessed by asking about specific symptoms of chronic illness (e.g., fever or diarrhea lasting ≥1 month), as well as by asking nine quality of life (QOL) questions related to perceived physical health; for example, if someone said they were bothered “some” or “very much” by physical problems related to their HIV, this was considered to represent a negative physical QOL response. Summary scores were created based on the number of negative QOL responses that were provided. Selected data were also abstracted from the participant’s HIV clinic record, including most recent CD4+ count, World Health Organization (WHO) clinical stage [23], and height and weight to calculate body mass index (BMI).

Participants were asked three questions about food insecurity in the past 3 months, such as whether they “sometimes” or “often” went to bed hungry, ate smaller or fewer meals, or went a whole day without eating because there was not enough food.

Participants were also asked whether they agreed or not with eight statements indicating social connectedness and social support from others, such as whether they had someone to talk to in times of stress. Summary scores were created based on the numbers of positive social support statements that were agreed to.

### Analysis

Confidence intervals (CI) are reported for selected proportions, standard deviation (SD) for mean values, and interquartile range (IQR) for median values. Predictors of both any khat use in the past year and heavy khat use were evaluated using Chi square for categorical variables, and *t* tests for continuous variables. Multivariate analysis was conducted using logistic regression. All data were double-entered; any discrepancies between entries were reconciled by checking the original data collection forms. All analyses were conducted using SAS version 9.3 (SAS Institute Inc., Cary, NC).

### Human subjects

Institutional Review Board approval was obtained from the University of Minnesota, and Federal Democratic Republic of Ethiopia’s Ministry of Science and Technology. Informed consent was obtained from all participants. For their time and effort, participants were reimbursed 70 Ethiopian birr (about $3.50 US) at the time of the survey.

## Results

### Study participants

Of 322 participants, 60% were from Dil Chora Hospital and 40% from Hiwot Fana. Characteristics of study participants are summarized in Table 1. Mean age was 33.8 years (range 18–70). Many participants had advanced clinical disease; 35% were WHO stage 3 or 4, and the median CD4+ count was 179 cells/mm^3^ (IQR = 90,346). The median BMI was 19.6 kg/m^2^ (IQR = 17.2, 22.0); 37% were ≤18.5 kg/m^2^, considered underweight.

**Table 1:** Characteristics of patients from Dil Chora (Dire Dawa) and Hiwot Fana (Harar) Hospital HIV clinics (N-322)

	Number and % of total^a^
*Gender*	
Male	145 (45%)
Female	177 (55%)
*Age (years)*	
≤25	52 (16%)
26–35	166 (52%)
>35	104 (32%)
*Marital*	
Single	79 (25%)
Married	114 (35%)
Widow	45 (14%)
Other (divorced, separated)	84 (26%)
*Education*	
No school	81 (25%)
Some primary school	108 (34%)
Above primary school	133 (41%)
*Religion*	
Orthodox Christian	195 (61%)
Muslim	101 (31%)
Protestant/other	26 (8%)
*Occupation*	
Day labor	113 (36%)
Housewife	42 (13%)
Business (e.g., merchant)	34 (11%)
Civil servant	28 (9%)
Other	50 (16%)
Unemployed	48 (15%)

^a^Missing data excluded from analysis

When asked to rate their overall health, 48% said very poor or poor. When asked 7 specific questions about symptoms over the past month, responses included chronic fatigue (71%), weight loss (58%), chronic pain (51%), chronic fever (40%), chronic cough (39%), chronic diarrhea (15%) and oral pain/sores (20%); 56% of patients had three or more of these symptoms. In response to nine questions about physical QOL, 86% gave a negative response to at least one QOL statement. For example 66% said they were bothered “some” or “very much” by physical problems related to their HIV, 64% said physical pain limited their activities, and 47% said they did not have enough energy for everyday life. The mean number of negative QOL statements agreed to was 4.8 (SD = 3.3).

Twenty-seven percent gave positive responses (“sometimes”/“often”) to one or more questions about food insecurity in the past 3 months; for example 19% reported sometimes or often going to bed hungry because there was not enough food. When asked 8 questions about social support or connectedness, 26% agreed with fewer than three positive statements. For example, 48% said they did not know of anyone who would help them if they needed it, and 38% said they did not have a feeling of closeness with anyone. The mean number of responses indicating positive social support was 4.9 (SD = 3.0).

### Khat use

Two hundred forty-two (75%) respondents (84% of men and 68% of women) reported ever using khat during their lifetime. Among lifetime users, age at first use was ≤12 years for 6%, 13–15 years for 16%, 16–18 years for 31%, and ≥19 years for 46%. Two hundred nine (65%, 95% CI 59–70%) respondents reported using khat within the previous year. Among those using khat in the previous year, the median number of days used in a typical month was 30 (daily use) (IQR = 10,30); during a typical day, khat was chewed a median of 5 h (IQR = 4,8). The median number of hours used in a typical month was calculated as 120 (IQR = 45,180 h). Sixty-nine respondents, or 21% (95% CI 17–26%) of all persons interviewed, reported using khat ≥180 h during a typical month.

Participants who used khat in the previous year were asked several questions concerning perceptions of khat’s health risks and their own drug use (Table 2). Three quarters believed khat use was dangerous for one’s health if one was HIV-positive, and 21% felt the need to cut down or control use but had difficulty doing so (Table 2).

**Table 2:** Attitudes about khat use among those Reporting use within the previous year, Dil Chora (Dire Dawa) and Hiwot Fana (Harar) Hospital HIV Clinics (N = 209)

	% responding affirmatively
Using khat is dangerous for your health if you have HIV	75
Using khat every day is dangerous for your health if you have HIV	84
Have you ever felt a need to cut down or control your khat use but had difficulty doing so?	21
Do you use khat within half an hour of waking up?	20
Do you find it difficult to not use khat in places where you shouldn’t, such as work, church, school, or a hospital?	9
If you are so sick that you are in bed most of the day, or if you are so sick that you have to cut back on your normal activities or routines, do you still use khat?	8

### Correlates of khat use in past year

By demographic characteristics, those who used in khat in the previous year were more likely to be male, Muslim and (of borderline significance) day laborers, and less likely to be married and Protestant (Table 3). Those using khat were also more likely to rate their health status as poor, and (of borderline significance) to have an underweight BMI. Khat users reported a mean of 3.1 chronic symptoms compared to 2.5 among non-users (p = 0.009), a mean of 4.9 negative QOL statements versus 4.5 among non-users (p > 0.10), and a mean of 4.8 positive social support statements vs. 5.0 among non-users (p > 0.10).

**Table 3:** Characteristics of patients from Dil Chora (Dire Dawa) and Hiwot Fana (Harar) Hospital HIV clinics, comparing those who did and did not use khat in the previous year, and comparing heavy khat users (≥180 h in a typical month) to all other participants: univariate analysis

	% Who used khat		% who used khat	
	In past year (%)	p value	≥ 180 h/month (%)	p value
*Gender*				
Male	75	<0.001	28	0.015
Female	56		16	
*Age (years)*				
≤25	62	0.519	19	0.556
26–35	63		20	
>35	69		25	
*Marital*				
Single	70	0.051	27	0.005
Married	56		11	
Other	70		27	
*Occupation*				
Day labor	73	0.054	25	0.491
Unemployed	60		23	
Other	60		19	
*Education*				
No school	59	0.197	27	0.281
Some primary school	71		18	
Above primary school	63		21	
*Religion*				
Orthodox Christian	63	<0.001	17	0.010
Muslim	76		31	
Protestant/Other	26		9	
*BMI (kg/m* ^*2*^ *)*				
≤18.5	71	0.066	29	0.011
>18.5	61		17	
*WHO stage*				
1–2	67	0.226	19	0.142
3–4	60		26	
*Health (self-rated)*				
Poor/very poor	71	0.039	26	0.030
Good/fair	60		16	
*Food insecurity (last 3 months)*				
Yes	72	0.123	23	0.728
No	62		21	

### Correlates of heavy khat use

Because khat use was relatively common in this population, we analyzed factors associated with the heaviest use of khat, defined as a calculated total of ≥180 h during a typical month, or the top quartile among current khat users. The comparison group was those with a calculated total of <180 h use in a typical month, including those who reported no use within the previous year.

By demographic characteristics, those who used khat >180 h in the past year were more likely than other participants to be male and Muslim, and less likely to be married and Protestant (Table 3). Heavy users were also more likely to rate their health status as poor and to have an underweight BMI. Heavy khat users reported a mean of 3.9 chronic symptoms compared to 2.7 among others (p < 0.001), a mean of 6.1 negative QOL statements vs. 4.4 among others (p < 0.001), and a mean of 4.4 positive social support statements versus 5.0 among others (p > 0.10).

Multivariate analysis was conducted to identify demographic predictors of heavy khat use, entering all variables shown in Table 4 into the model. Factors independently associated with heavy khat use included male gender [odds ratio (OR) 2.36, 95% CI 1.23, 4.52] and Muslim religion (OR 2.55, 95% CI 1.34, 4.87); those who were married were less likely to report heavy use (OR 0.38, 95% CI 0.19, 0.77) (Table 4).

**Table 4:** Demographic characteristics of patients from Dil Chora (Dire Dawa) and Hiwot Fana (Harar) Hospital HIV Clinics, comparing heavy khat users (≥180 h in a typical month) to all other participants: multivariate analysis

Demographic characteristic	Odds ratio (95% CI)	p value^a^
*Gender*		
Male (vs. female)	2.36 (1.23, 4.52)	0.010
*Age*		
≤25 years (vs. >35)	1.03 (0.42, 2.54)	0.978
26–35 years (vs. >35)	1.07 (0.55, 2.08)	
*Marital*		
Married (vs. other)	0.38 (0.19, 0.77)	0.007
*Religion*		
Muslim (vs. Orthodox Christian)	2.55 (1.34, 4.87)	0.009
Protestant (vs. Orthodox Christian)	0.55 (0.12, 2.57)	
*Employment*		
Day laborer (vs. other occupation)	1.33 (0.68, 2.60)	0.704
Unemployed (vs. other occupation)	1.20 (0.51, 2.84)	
*Education*		
No school (vs. above primary)	1.22 (0.56, 2.66)	0.413
Some primary school (vs. above primary)	0.73 (0.35, 1.51)	

^a^For overall demographic characteristic

### Other substance use

During the previous year, 30% of respondents reported smoking cigarettes or shisha (tobacco smoked with a hookah), and 43% reported drinking alcohol. Tobacco use in the past year was reported by 46% of those who also used khat in the previous year, compared to only 1% of those who did not use khat (p < 0.001). Those who used khat in the previous year reported smoking a mean of 5 cigarettes/day. Heavy khat users reported a mean of smoking 8 cigarettes/day.

Alcohol use in the previous year was reported by 49% of those reporting khat use in the previous year, compared to 31% of those who did not use khat (p = 0.002). Those who used khat in the previous year reported a mean of 36 drinks/month, compared to 11 drinks/month among those who did not use khat (p < 0.001). Heavy khat users reported a mean of 51 drinks/month.

## Discussion

Among HIV patients newly enrolled in care at two large Ethiopian hospitals, we found a high prevalence of khat use, with 75% reporting lifetime use and 65% reporting use within the previous year. Among those using khat, over half reported chewing khat every day, and almost two-thirds used khat ≥5 h/day. Use of khat in the past year was also associated with poorer self-reported health status, a lower BMI, and a greater number of chronic symptoms. Heavy users (≥180 h in a typical month) reported all of these undesirable clinical outcomes, as well as a greater number of negative physical QOL statements.

Our study found that most persons who ever used khat had used it during the year prior to enrollment in care; future analyses in this prospective study will compare khat use in the year before and after enrollment. Studies of khat have used different time frames to estimate recent use, such as past month [11] or past year [12]. Although we used past year, data from the 2011 Ethiopian Demographic and Health Survey using a 30 day time frame are consistent with our conclusions that most persons ever using khat have used it recently. Among men and women from the Harari region who ever used khat, 91 and 88% respectively had used one or more times it in the last 30 days; among men and women from Dire Dawa, corresponding rates were 85 and 96% [19].

HIV patients who reported khat use in the past year, especially heavy use, reported a greater number of chronic symptoms and a more negative physical QOL. Whether khat users who have such symptoms are more likely to use khat to help alleviate them, or whether chronic and habitual use causes increased symptomatology is unclear. However, other studies also report greater physical illness in khat users [6] and habitual khat use has been shown to be associated with a wide variety of physical and mental health harms, including cognitive impairment that may affect daily life activities [1–5, 24].

Khat users in our study were also significantly more likely to both use tobacco and drink alcohol. The association of khat use with both smoking and alcohol use has been reported from other studies as well [10, 11, 25]. We cannot say whether khat consumption predisposes users to use of these other substances (for example, use of alcohol to counteract stimulant properties of khat), or whether this association reflects underlying stressors or psychological factors that cause people to use multiple substances. Since both alcohol abuse and smoking can have negative health outcomes for HIV patients [14, 15], use of multiple substances may add to the adverse health effects potentially associated with khat.

Although khat is legal in Ethiopia and may be considered part of the lifestyle in some countries, it also has a potential for development of dependence [1, 3, 26, 27]. Although most respondents believed khat use (especially every day) was dangerous for health of HIV-infected individuals, use on a daily basis was common; 21% of current users reported that they felt a need to cut down or control their khat use but had difficulty doing so.

Our results have several implications for strategies to prevent or reduce khat use. Over half (54%) of khat users reported first use at age 18 years or younger. Given the prevalence of khat use in some populations of young people [8, 9], this supports the importance of drug education programs in schools and other settings where youth congregate. In addition to being a major cash crop, in certain communities khat has a social and cultural significance and acceptance [26, 28]. Rather than advocating for abstinence in all populations, a more balanced harm reduction approach that discourages excessive khat use or onset early in life may be more reasonable and favorable received [28].

Our study has several limitations. First, we chose for this study two cities where khat use is especially common; persons in different geographic areas may have a different prevalence and correlates of use. Second, khat use was self-reported, and some persons may have underreported their frequency of use. Third, associations between khat use and other factors in this survey do not necessarily imply temporality or causality. Finally, during khat sessions, leaves are kept in the mouth and typically chewed over several hours to release active components, and different khat plants may contain different concentrations of active cathinone products; therefore, the number of hours chewed may not strictly correspond with number of leaves ingested. As a statistical threshold, we chose the top quartile of hours khat was used in a typical month as the cut-point for heavy use. However, future studies, ideally in correlation to cathinone blood levels, can help to clarify how frequency and amount of use is associated with drug absorption and specific biological harms.

## Conclusion

Among HIV positive Ethiopians entering care in two large eastern HIV clinics, two-thirds reported khat use within the past year, with a majority of these using khat on a daily basis. Use of khat, especially among heavy users, was associated with a number of measures of poorer physical health, including a greater number of chronic symptoms and poorer self-reported health status. In a setting where khat use is legal and widely utilized, defining patterns of responsible use and developing community-based interventions represent an important health priority to be included as part of a comprehensive care package for people living with HIV.

## Abbreviations

BMI: body mass index
CI: confidence intervals
HIV: human immunodeficiency virus
IQR: interquartile range
QOL: quality of life
SD: standard deviation
WHO: World Health Organization
